# Chromosome-level genome assembly of *Tadehagi triquetrum* provides new insights into genome evolution and biosynthesis of tadehaginoside

**DOI:** 10.1016/j.pld.2025.12.017

**Published:** 2026-01-06

**Authors:** Yongjian Luo, Ru Wang, Daoshou Qiu

**Affiliations:** a Key Laboratory of Crops Genetics and Improvement of Guangdong Province, Crops Research Institute, Guangdong Academy of Agricultural Sciences, Guangzhou 510640, China; b Key Laboratory of Forestry Biotechnology of Hunan Province, Central South University of Forestry and Technology, Changsha 410004, Hunan, China; c Faculty of Landscape Architecture and Horticulture, Southwest Forestry University, Kunming 650224, China

**Keywords:** *Tadehagi triquetrum*, Telomere-to-telomere genome, BADH gene family, Tadehaginoside, Flavonoid biosynthesis, Phylogenetic analysis

## Abstract

• First chromosome-level genome of *Tadehagi triquetrum* (627.33 Mb, contig N50 = 34.04 Mb, 99.2% BUSCO).
• Phylogenomics places *T. triquetrum* sister to soybean, diverged ～34.16 Mya.
• Identification and characterization of TtHCT1 reveals tadehaginoside biosynthetic step.

*Tadehagi* *triquetrum* is a shrub or subshrub of the legume family (Fabaceae), subfamily Papilionoideae, characterized by winged leaves resembling a gourd shape (Fang et al., 2025). It is primarily distributed in southern China, including Yunnan, Guangdong, Guangxi, and Hainan. In the Lingnan region, *T. triquetrum* is commonly used in traditional Chinese medicine, with the entire plant and its roots serving as medicinal material. These plant materials are used both as single herbs and in compound formulations to treat a variety of diseases, including colds, hookworm infections, lung abscesses, and other types of inflammation (Tang et al., 2022). Previous studies have identified over 70 secondary metabolites from *T. triquetrum*, including flavonoids, phenylpropanoids, phenolic compounds, triterpenoids, and steroids (Tang et al., 2022). Notably, the major bioactive component of *T. triquetrum* is a phenylpropanoid compound named tadehaginoside (Zhang et al., 2016). Tadehaginoside has been shown to exhibit broad biological activities, with potential therapeutic effects for metabolic diseases such as obesity, diabetes, and atherosclerosis (Tang et al., 2014; Zhang et al., 2015; Zhao et al., 2021). However, several factors have hindered chemical synthesis of tadehaginoside, including its low content in plants and the high cost of extraction. We reasoned that a simpler and more efficient approach to production of tadehaginoside is biosynthesis (Tian et al., 2024). Unfortunately, the key biosynthetic pathways and core genes of tadehaginoside have yet to be identified. On the other hand, the Fabaceae family is one of the 34 largest families of flowering plants, comprising 765 genera and nearly 20,000 species worldwide (Zhao et al., 2021). Although genome data for 413 Fabaceae plants have been completed, this still covers less than 1% of the species in family (144 species) (Yu et al., 2025). Sequencing new species and their genomes helps expand our understanding of biodiversity, species evolution, and ecosystem functions (Davis and Knapp, 2025; Zhang et al., 2025a, b). The taxonomic position of the *Tadehagi* genus has long been controversial, mainly due to its morphological similarity to the *Desmodium* genus (Fang et al., 2025). Jabbour et al. (2018) reconstructed the phylogenetic relationships within the Fabaceae tribe using chloroplast DNA fragments (*rbcL*, *psb*A-*trn*H) and nuclear gene sequences (nrITS-1). Their findings support classifying *Tadehagi* as an independent genus. However, the phylogenetic tree topology constructed from these two datasets shows significant inconsistencies, indicating that more genetic data are needed to provide new insights into species evolution. To identify key genes involved in the biosynthesis of tadehaginoside, we analyzed genomic, transcriptomic and metabolomic data of *T. triquetrum*. For this purpose, we created a chromosomal-level genome assembly of *T. triquetrum*, a first for the species.

The plant species *Tadehagi triquetrum* used in this study was a wild local variety collected from Baiyun Mountain in Guangzhou, Guangdong Province, China (23.17° N, 113.29° E) (Fig. 1A). To estimate the preliminary characteristics of the *T. triquetrum* genome, we generated 18.35 Gb of high-accuracy HiFi data using the PacBio platform, with an average read length of 13.792 kb. Our K-mer analysis (K = 21) indicated that the plant exhibits a high level of heterozygosity and genomic repetitiveness, with an estimated genome size of approximately 520.06 Mb and a heterozygosity rate of about 0.18 % (Fig. 1B). Subsequently, we assembled these HiFi reads into a 627.33 Mb draft genome using Hifiasm, which consists of 33 contigs (N50 = 34.03 Mb). We then generated 97.23 Gb of Hi-C paired-end sequencing data for *T. triquetrum* and anchored the 33 contigs onto 10 chromosomes (Fig. 1C and D). After multiple rounds of assembly, error correction, alignment, extension, and gap filling, we obtained 10 scaffolds, which included 22 gaps, primarily due to the lack of sequencing coverage (Table S1). The N50 of the scaffolds was 64.98 Mb, with the longest scaffold measuring 82.85 Mb (Fig. 1D). We also examined the telomeres of the genome and found that, except for chromosomes 1 and 4, which each have a single telomere, chromosomes 3, 7, 8, and 10 are complete chromosomes, with telomeres at both ends and no gaps. Other chromosomes also have intact telomeres at both ends, featuring the characteristic AAACCCT sequence repeat (Table S1). PacBio HiFi reads map to the genome with an exceptionally high rate of 99.94%. The BUSCO and quality value (QV) assembly scores of our genome are 99.2% and 68.94, respectively, which are comparable to those of several well-assembled end-to-end genomes (Table S2). For instance, the common bean genome has a BUSCO completeness of 99.5% and a QV of 54.86, while Medicago exhibits a BUSCO score of 99.32%. Additionally, the BUSCO scores of *Bauhinia purpurea* and *B. variegata* are 99.0% and 99.2%, respectively. These results provide strong evidence for the high fidelity and completeness of our assembled *T. triquetrum* genome.

**Fig. 1:**
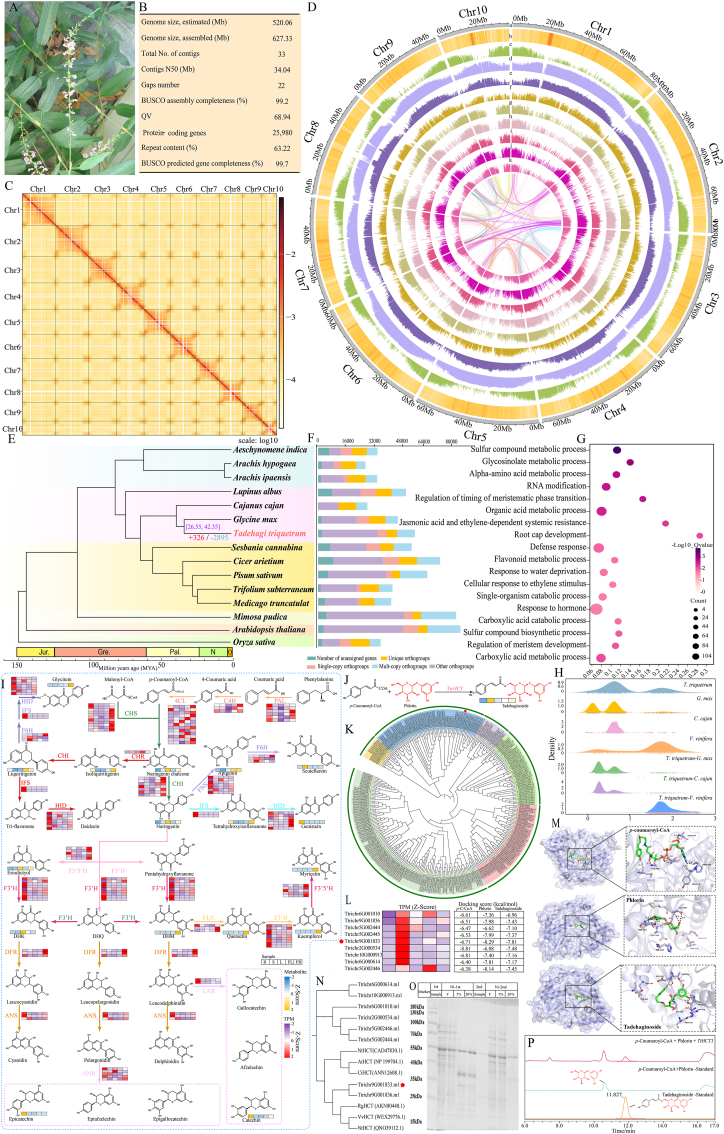
**Chromosome-level genome assembly and analysis of *Tadehagi triquetrum*. A.** Phenotypic characteristics of *T. triquetrum* samples. **B.** Genome assembly statistics. **C.** Hi-C contact matrix analysis of the *T. triquetrum* genome. The heatmap illustrates the high resolution and completeness of the independently assembled chromosomes. **D.** Overview of the genomic features of the *T. triquetrum* genome. From outer to inner circles: (a) chromosome length, (b) GC content within 100 kb sliding windows, (c) gene density within 100 kb sliding windows, (d) transposable element (TE) repeat density within 200 kb sliding windows, (e) Gypsy repeat density within 200 kb sliding windows, (f) Copia repeat density within 200 kb sliding windows, (g–k) gene expression levels (Transcripts Per Million, TPM) in roots, stems, leaves, flowers, and fruits within 200 kb sliding windows. **E.** Phylogenetic tree depicting the divergence between Miconioides and 12 other plant species. The expansion and contraction of gene families are indicated in red and green, respectively. The numbers on the timeline represent the divergence time of the species (million years ago, MYA). **F.** Distribution of orthologous and paralogous gene families among 15 plant species. **G.** Gene Ontology (GO) biological function annotation analysis of expanded gene families. **H.** Ks distribution of homologous genes between *T. triquetrum* and other species. **I.** Flavonoid biosynthesis pathway analysis in *T. triquetrum.***J.** Predicted biosynthetic pathway and content of tadehaginoside. **K.** Identification and phylogenetic analysis of the BADH gene family in *T. triquetrum*, soybean, and *Arabidopsisthaliana*. **L.** Identification of key genes correlated with tadehaginoside content in the BADH gene family and molecular docking affinity coefficients. **M.** Molecular docking interactions between *TtHCT1* (Ttrichr9G001833) and *p-*Coumaryl-Coenzyme A, Phlorin, and Tadehaginoside. **N.** Phylogenetic tree of nine core HCT genes constructed with those from other species. **O.** Protein expression and purification of *TtHCT1*. **P.** In vitro enzyme activity assay and validation of *TtHCT1* using standard *p*-Coumaryl-Coenzyme A, Phlorin, and tadehaginoside in HPLC. *TtHCT*1-pET32 (+) empty vector protein was used as a negative control.

The genome consists of 63.22% repetitive sequences, with the majority being long terminal repeat (LTR) sequences, accounting for 47.46% of the genome. DNA transposons, long interspersed nuclear elements (LINEs), and simple repeats account for 3.03%, 0.17%, and 1.22% of the genome, respectively (Table S3). To facilitate genome annotation of *T. triquetrum*, RNA sequencing was performed on root, stem, leaf, flower, and pod tissues, generating a total of 135.25 Gb of clean reads. A total of 25,980 protein-coding genes were identified through a combination of de novo prediction and homologous annotation strategies, with an average coding sequence length of 4541 bp. Each gene contains an average of 8.36 exons (Fig. 1D). Of these, 23,775 genes were annotated with at least one data source (KEGG, GO, COG, PFAM, and NT). BUSCO evaluation of the predicted gene set revealed 99.7% completeness, with only 0.3% of genes missing, indicating the robustness of the gene annotation (Table S4).

To investigate the phylogenetic position of *Tadehagi triquetrum*, we selected 13 species from the subfamily Papilionoideae, along with the outgroups *Oryza sativa* and *Arabidopsis thaliana*. Using OrthoFinder, we clustered all protein sequences into 43,463 orthologous gene families (OGs) (Fig. 1F). The phylogenetic tree based on single-copy OGs revealed that *T. triquetrum* is closely related to soybean, diverging approximately 34.16 million years ago (MYA) (Fig. 1E). This relationship further supports its evolutionary position within the Papilionoideae subfamily of the Leguminosae. To explore the dynamics of gene families, we identified 326 gene families that were expanded in *T. triquetrum*, while 2895 gene families were contracted. These 326 expanded OGs contained 1583 genes (Table S5). Gene Ontology (GO) enrichment analysis of the expanded gene families showed significant enrichment in biological processes such as sulfur compound metabolism, “glycosinolate metabolism, jasmonic acid and ethylene-dependent systemic resistance, defense responses, and single-organism catabolism (Fig. 1G). Analysis of the synonymous substitution rate (*K*s) distribution between syntenic gene pairs revealed that *T. triquetrum* underwent two rounds of whole-genome duplication (WGD) events (Fig. 1H). It first experienced an ancient whole-genome triplication event (γ event) shared by core eudicots, represented by *Vitis vinifera*. Secondly, *T. triquetrum* shared an ancestral Papilionoideae-wide genome duplication event (PWGD) with soybean and *Cajanus cajan* (Luo et al., 2024).

Studies have shown that flavonoid/isoflavonoid biosynthesis genes, acquired through gene duplication in legumes, play a crucial role in the evolution of root nodule symbiotic nitrogen fixation (RNS) (Liu et al., 2024). In this study, we employed a widely targeted metabolomics approach to profile the accumulation patterns of flavonoid compounds in various tissues of *Tadehagi triquetrum* for the first time. Our results identified 200 flavonoid compounds in *T. triquetrum*, most of which are glycosylated (Table S6). Further analysis indicated that certain flavonoid metabolites are primarily enriched in the fruit and root tissues, including taxifolin 7-O-rhamnoside, catechin, astragalin, and nicotiflorin, which are highly concentrated (greater than 2.0 mg/g) in the fruit (Table S6). Next, we analyzed the biosynthetic pathways of flavonoid compounds and their gene expression patterns in *T. triquetrum*. We found that the relevant genes are predominantly expressed in the roots and fruits. This result is consistent with the flavonoid accumulation patterns observed in the metabolomics analysis (Fig. 1I and Table S7). The accumulation of flavonoids in the roots likely contributes to the formation of root nodules, while their accumulation in fruits may be associated with functions such as UV radiation protection, antioxidation, and pest resistance (Nabavi et al., 2020; Bosse et al., 2021; Patil et al., 2024; Ren et al., 2024). HPLC quantification of the tissue-specific distribution of tadehaginoside, indicated that concentrations were highest in the stems, reaching 11.1 mg/g (Root: 0.0388 mg/g, Stem: 11.1009 mg/g, Leaf: 3.1242 mg/g, Fruit: 1.7226 mg/g). Based on the chemical structure of tadehaginoside (Ban et al., 2025), we hypothesized that its key biosynthetic precursors are p-Coumaryl-Coenzyme A and Phlorin (Fig. 1J). Studies have shown that p-Coumaryl-Coenzyme A can be acylated by the Hydroxycinnamoyl Transferase (HCT) acyltransferase subfamily of the BADH gene family, which contains the HXXXD and DFGWG domains (Tian et al., 2021; Yang et al., 2023; Ban et al., 2025; Huang et al., 2025). This allowed us to identify 105 BADH gene family members in the *T. triquetrum* genome (Fig. 1K). Gene expression analysis identified nine BADH genes co-expressed with tadehaginoside (Ttrichr6G001010, Ttrichr9G001836, Ttrichr5G002444, Ttrichr5G002445, Ttrichr9G001833, Ttrichr2G000534, Ttrichr10G000913, Ttrichr6G000614, Ttrichr5G002446) (r^2^ > 0.9, *p* < 0.05) (Fig. 1L and Table S8).

To predict the binding energies of candidate genes in the tadehaginoside biosynthetic pathway, we performed molecular docking of these nine BADH genes with *p*-Coumaryl-Coenzyme A, Phlorin, and tadehaginoside. The *TtHCT*1 (Ttrichr9G001833) gene exhibits a strong binding affinity for *p*-Coumaryl-Coenzyme A, Phlorin, and tadehaginoside, with binding energies of 6.71, −8.44, and 8.28 kcal/mol, respectively, suggesting its potential role in synthesizing tadehaginoside (Fig. 1L). Detailed molecular docking simulations reveal that these compounds bind within the same domain, with key amino acid residues in *TtHCT*1 (Gly-299, Thr-173, Asp-380) involved in the binding sites for *p*-Coumaryl-Coenzyme A, Phlorin, and tadehaginoside (Fig. 1M). Phylogenetic analysis indicates that TtHCT1 shares a close relationship with some HCT sequences known to bind *p*-Coumaryl-Coenzyme A and glycosides (Fig. 1N). Correlation analysis, molecular docking, and phylogenetic analysis all indicate that *TtHCT*1 is a key enzyme in the biosynthesis of Tadehaginoside. To investigate the catalytic function of the protein encoded by the *TtHCT*1 gene, the full-length *TtHCT*1 gene was inserted into the pET32a expression vector with a His tag, and protein expression was induced overnight with IPTG. The recombinant protein was purified using a nickel column, and its molecular weight was determined to be approximately 51.68 kDa, which closely matched the theoretical value (Fig. 1O). To verify the catalytic function of the *TtHCT*1 recombinant protein, we incubated the *TtHCT*1 recombinant protein with p-Coumaryl-Coenzyme A and Phlorin. HPLC analysis indicate that *TtHCT*1 recombinant protein catalyzes p-Coumaryl-Coenzyme A and Phlorin into tadehaginoside (Fig. 1P). This indicates that the *TtHCT*1 recombinant protein has the catalytic ability to synthesize tadehaginoside.

In summary, our assembly of a chromosome-level genome for *Tadehagi triquetrum* has helped confirm that a BADH gene named *TtHCT*1 catalyzes precursor metabolites p-Coumaryl-Coenzyme A and Phlorin into tadehaginoside. This finding is an important step in the eventual biosynthetic production of tadehaginoside, a compound that may be used to treat obesity, diabetes, and atherosclerosis.

## Data availability statement

All the genome assembly files generated in this study have been deposited in the National Center for Biotechnology Information (NCBI) Sequence Read Archive (SRA) (https://www.ncbi.nlm.nih.gov/sra/) under BioProject accession number PRJNA1347618. The genome assembly and annotation files have been deposited in Science Data Bank (Science DB: https://scidb.cn/en/c/pd) under accession https://doi.org/10.57760/sciencedb.j00143.00135.

## CRediT authorship contribution statement

**Yongjian Luo:** Writing – original draft, Formal analysis, Methodology, Writing – review & editing, Data curation, Investigation, Visualization. **Ru Wang:** Resources, Writing – review & editing, Formal analysis, Validation. **Daoshou Qiu:** Project administration, Conceptualization, Funding acquisition, Writing – review & editing, Supervision.

## Declaration of competing interest

The authors declare that they have no known competing financial interests or personal relationships that could have appeared to influence the work reported in this paper.
