# Supplementary material for: Chromosome-level genome assembly of Tadehagi triquetrum provides new insights into genome evolution and biosynthesis of tadehaginoside
Source: Plant Divers. 2026 Jan 6;48(4):846–50. doi: 10.1016/j.pld.2025.12.017 (PMC13424416; doi:10.1016/j.pld.2025.12.017)
Supplement: Multimedia component 1 [file mmc1.docx]

**Supplementary Materials and Methods**

# **Plant Material, DNA Extraction, and Sequencing**

The *T. triquetrum* plant used in this study was originally collected from a spice plantation in Lufeng City, Guangdong Province, China (35°2' N, 104°3' E) and subsequently relocated to the Guangdong Provincial Crop Germplasm Resource Nursery (23°16' N, 113°27' E) for long-term maintenance under natural conditions. The sample ID is GD2021441581. All genomic sequencing samples were derived from fresh leaves of a single plant. For long-read sequencing, a PacBio SMRTbell library was constructed using the SMRTbell Prep Kit 3.0 (Pacific Biosciences, Menlo Park, CA, USA) according to the manufacturer's protocol. Sequence was performed on the PacBio Revio platform (PacBio Sequel II system) to generate high-fidelity (HiFi) data. To improve the continuity of the genome assemblies, we created Hi-C libraries for the young leaves of the same plant using the GenSeq Hi-C library preparation kit (Cloud-Seq Bio Co., Ltd., China, Shanghai). The kit involves crosslinking, DpnII digestion, biotin labeling, ligation, shearing, and biotin capture (Zhang et al., 2017). Sequencing of the Hi-C fragments was conducted on the DNBSEQ-T7 platform (MGI, Shenzhen, China).

# **De novo Genome Assembly**

First, HiFiAdapterFilt v3.0.0 (Sim et al., 2022) was used to remove reads with residual PacBio adapter sequences from the HiFi sequencing data and perform quality control. Jellyfish v2.3.1 (Marçais and Kingsford, 2011) was used to count k-mers in the HiFi sequencing data with the parameter kmer=21. Genomescope.R v2.0 (Vurture et al., 2017) was employed to estimate the genome size, setting the maximum k-mer coverage threshold to 10,000. The initial genome assembly of PacBio Revio sequencing platform HiFi reads was performed using Hifiasm v0.15.5 (Cheng et al., 2021) with default parameters. Subsequently, the genome was polished three times using Pilon v1.24 (Walker et al., 2014). Redundant overlapping contigs were removed using Purge_Haplotigs v1.1.2 (Roach et al., 2018). The final round of gap filling for the draft genome was conducted using NextDenovo v2.17-r941 (Hu et al., 2024) and TGS_Gapcloser2 v1.9.4 (Xu et al., 2020). Next, the Hi-C reads were aligned to the assembled genome using Juicer v1.60 (Durand et al., 2016) software, allowing us to obtain interaction data between chromatin regions across contigs. The 3D-DNA v180922 (Loescher et al., 2018) pipeline was used to automatically generate candidate chromosome-level assemblies to correct erroneous connections, orders, and orientations, and to organize the contigs into the draft chromosome assembly. Errors in the chromosome-level assembly were manually corrected in Juicebox v2.20.00 (Robinson et al., 2018), and Hi-C reads were aligned to the chromosome-level assembly using HiCExplorer v3.7.2 (Wolff et al., 2020) to evaluate the clarity of structural domains across different chromosomes. The final chromosome-level genome assembly was assessed using BUSCO v3.1.0 (Simão et al., 2015) based on the embryophyta_odb10 gene set. Minimap2 v2.28 (Li, 2018) was used to map PacBio HiFi reads to the assembled genome to evaluate coverage and average depth. Genome quality was assessed using LTR_retriever v2.9.681 (Ou and Jiang, 2018). Finally, Mequery v1.3 (Rhie et al., 2020)was used to evaluate the assembly's overall quality (QV) and completeness.

# **Genome Annotation**

The identification of known TEs in the genome is performed using RepeatMasker v4.1.0 (Tarailo-Graovac and Chen, 2009) with the Repbase TE library, and the TE protein database is queried using RepeatProteinMask. Subsequently, RepeatModeler2 v1.0.11 (Flynn et al., 2020) is employed to construct de novo repeat libraries for each genome, followed by detailed analysis, refinement, and classification. LTRs are identified with LTR_Retriever v2.9.681 (Ou and Jiang, 2018) and further classified using TESorter v1.4.682 (Zhang et al., 2022). The terminal regions of LTRs are aligned with Mafft v7.50584 (Katoh and Standley, 2013), and insertion times for LTRs are calculated using the formula T = K/2r, where K represents the genetic distance, and r is the substitution rate of 1.3e−8 substitutions per site per year. An unrooted phylogenetic tree is constructed using the neighbor-joining method with FastTree v2.1.1185 (Price et al., 2010). For accurate protein-coding gene prediction, a combination of transcriptomic, homology-based, and de novo approaches is used. De novo predictions are made with Augustus (Gabriel et al., 2024), GenScan (Burge and Karlin, 1997), and GlimmerHMM (Majoros et al., 2004), utilizing the Arabidopsis training set. Homology-based predictions employ GeMoMa (Keilwagen et al., 2019), incorporating protein sequences from *P. nigrum* and other species. Transcriptomic predictions align the de novo transcriptome assembly with the genome and refine gene structures using PASA. Gene model consensus sets are generated using EVidenceModeler (Haas et al., 2008), excluding single-exon genes supported solely by transcriptomic data and genes with fewer than three exons. To further enhance the reliability of annotations, TransposonPSI v 2.2.26 (Riehl et al., n.d.) is used to identify transposon-related genes, while Pseudogene Pipeline identifies pseudogenes. Pseudogenes and transposon-related genes with FPKM < 1 are excluded. Gene function annotations are performed using EggNOG-mapper (Cantalapiedra et al., 2021), Diamond, and InterProScan, providing annotations for GO, EC, KEGG pathways, COGs, and other functional categories. Non-coding RNA annotations are performed using Infernal v1.1.4 and tRNAscan-SE v2.0.9 (Chan and Lowe, 2019). The integrity of the genome or protein sequences is assessed using BUSCO (Simão et al., 2015) with the embryophyta_odb10 plant reference set.

# **Phylogenetic Analysis**

The longest protein sequences from each gene model of 15 plant species were extracted and subjected to an all-versus-all Diamond alignment. Homologous genomes were identified using the default parameters of OrthoFinder v3.0.1b1 (Emms and Kelly, 2019) software. The aligned tandem amino acid sequences were further refined using MAFFT v7.271 (Katoh and Standley, 2013), followed by trimming with trimAI v1.4.rev22. A maximum likelihood phylogenetic tree was constructed using RAxML v8.2.12 (Stamatakis, 2014) with the PROTGAMMAJTT model and 1,000 bootstrap replicates, using *A. trichopoda* as the outgroup. The species tree topology was calibrated using MCMCtree v4.9 (Puttick, 2019), incorporating two temporal calibration points: Node 1 (the common ancestor of *A. trichopoda* and *O. sativa*) estimated to be between 142.1 and 163.5 MYA; Node 2 (*Glycine max* and *T. triquetrum*) estimated to be between 21.8 and 50.5 MYA, with data sourced from the TimeTree (Kumar et al., 2022) database. A log-normal independent molecular clock model was used to generate 1,000,000 samples, discarding the first 25% as burn-in. The MCMCtreeR R package was employed to visualize the reliable divergence time intervals of each node in the species tree. Gene family expansion and contraction were assessed using CAFE v4.2.1 (Mendes et al., 2021), and conditional P-values for each gene family were calculated. Gene families with P-values < 0.05 were considered to have significantly accelerated rates of expansion or contraction. Gene families with more than 100 copies in a single species were removed. Homologous protein sequences between species were aligned using Diamond v0.9.29 (Buchfink et al., 2015) (E-value < 1e−5, C-score > 0.5), and syntenic blocks were identified using MCScanX v1.0.0 (Wang et al., 2012). To investigate the evolution of the *T. triquetrum* genome, the synonymous substitution rate (Ks) for collinear gene pairs was calculated using wgdi v1.1.1 (Sun et al., 2022).

# **Transcriptome and Metabolomic Analysis**

The transcriptome sequencing was performed on five different tissues, including roots, stems, mature leaves, flowers, and fruits. Each sample consisted of three biological replicates. Total RNA was extracted using the Trizol reagent kit (Invitrogen, Carlsbad, CA, USA), and its quality was assessed using the Agilent 2100 Bioanalyzer. Eukaryotic mRNA was enriched with Oligo(dT) beads, while prokaryotic mRNA was enriched by removing rRNA using the Ribo-ZeroTM Magnetic Kit (Epicentre, Madison, WI, USA). The cDNA libraries were constructed using the NEBNext UltraTM RNA Library Prep Kit for Illumina (New England BioLabs, Beijing, China). The resulting cDNA library was sequenced on the MGI T7 platform (MGI, Shenzhen, China) using a 2 × 150 bp paired-end run, with the experimental procedures following standard plant RNA extraction protocols. Sequencing was performed by Yuda Biotechnology Co., Ltd (Guangzhou, China). After filtering low-quality reads with fastp v0.23.4 (Chen et al., 2018), clean reads were mapped to the *T. triquetrum* genome using HISAT2 v.2.2.1 (Kim et al., 2015). Reads were quantified using featureCounts. Reads were quantified using featureCounts, and gene expression values were represented as TPM. The clusterProfiler v4.0 (Wu et al., 2021) package was used to perform GO and KEGG enrichment analysis on the genes in the identified tissue-specific modules.

The samples of biological materials preserved in ultra-low temperature cryopreservation were removed, and the samples were vacuum freeze-dried. A grinder (MM400, Retsch) was used to grind the samples for 1.5 min at 30 Hz with a final particle size of 5 μm, and 100 mg of powder was weighed. One mL extraction solution was used to dissolve 100 mg of powder. The samples were left at 4 °C for 12 h, during which time they were vortexed three times for a more complete extraction. After extraction, the samples were centrifuged (at 10,000 g) for 10 min and the supernatant was filtered through a microporous membrane (0.22 μm pore size) and stored in a vial for subsequent LC-MS/MS analysis. Aliquots of sample extracts were also taken and mixed to make quality control samples to verify their reproducibility under the same treatment. To ensure reproducibility, one QC sample is inserted into every 10 samples analyzed for the assay. The Ultra Performance Liquid Chromatography (Shim-pack UFLC SHIMADZU CBM20A) and Tandem mass spectrometry (UPLC-MS/MS) (Applied Biosystems 4500 QTRAP) were used to determine the content of flavonoids according to past research (Hou et al., 2021). In the API 4500 QTRAP LC/MS/MS system, the main parameters of the linear ion trap and triple quadrupole include electrospray ionization at 550 °C, mass spectrometry at 5500 V, curtain gas at 25 psi, and collision-activated dissociation at high. The collision-activated dissociation parameters were set to high. In the triple quadrupole, each ion pair is scanned and detected according to the optimized decluttering potential and collision energy. The data obtained were processed using the software Analyst 1.6.1 (AB SCIEX).

# **Flavonoid Biosynthesis Gene Identification and Molecular Docking**

To identify candidate genes involved in the biosynthesis pathway of alkaloids, BLAST searches were performed on the *T. triquetrum* genome assembly with an E-value threshold of ≤ 1e-5, using protein sequences from *A. thaliana* and *T. triquetrum* as queries. The enzymes and flavonoid compounds are abgreviated as follows: PAL, phenylalanine ammonia lyase; C4H, cinnamic acid 4-hydroxylase; 4CL, 4-coumarate; CHS, chalcone synthase; CHR, chalcone reductase; CHI, chalcone isomerase; F6H, flavonoid 6-hydroxylase; IFS, isoflavone synthase; HID, hydroxy isoflavanone dehydratase; FNS, flavone synthase; F6H, flavonoid 6-hydroxylase; F3’5′H, flavonoid 3′,5′-hydroxylase; F3’H, flavonoid 3’-hydroxylase; F3H, flavonoid 3-hydroxylase; DHK, dihydrokaempferol; DHQ, dihydroquercetin, DHM: dihydromyricetin; FLS, flavonol synthase; DFR, dihydroflavonol 4-reductase; ANS, anthocyanidin synthase; LAR, leucoanthocyanidin reductase; ANR, anthocyanidin reductase.

To identify BAHD proteins in *P. sarmentosum*, we downloaded the Pfam seed files for BAHD genes (PF02458, PF07247) from the Pfam database (<http://pfam.xfam.org/>). HMMER v3.0 (Potter et al., 2018) and local BLAST were employed to search for sequences containing BADH protein domains. The identified protein sequences were aligned to remove redundant sequences. A hidden Markov model (HMM) was then reconstructed for the detected proteins, and sequences with corresponding Pfam domains were searched again. Further validation of BAHD family members was performed using the online tools SMART and CDD. AlphaFold 3 performs homology modeling of the mutated sequences of BAHD. The best model is selected based on molpdf, DOPE, and GA341 potential scores. The geometric structure and quality of the model are checked online (<http://servicesn.mbi.ucla.edu/SAVES/>). Docking analysis is carried out using AutoDock Vina, and the results are analyzed using PyMOL software.

# **Prokaryotic expression and purification of *TtHCT1* proteins and In vitro enzyme assay**

To express *TtHCT1* (Forward Primer: ATGGCATACAAATACACC; Reverse Primer: ATGCATAGTTTTGTTCCTTTC) protein in E. coli, the *TtHCT1* ORF was cleaved using restriction enzymes and cloned into the pET32 vector. The recombinant plasmid was transformed into E. coli DE3 cells. After PCR screening for positive clones, cells were cultured in LB medium with 50 μg/mL ampicillin at 37°C, shaking at 180 rpm, until OD600nm reached 0.6-0.8. Protein expression was induced by adding 1 mM IPTG, and cells were incubated at 18°C with shaking at 160 rpm for 4 hours. After induction, cells were harvested by centrifugation, resuspended in 0.3 M NaCl, 50 mM HEPES buffer, and disrupted by sonication. The lysate was centrifuged, and the supernatant was filtered for purification. The supernatant (20 mL) was purified using a Ni-NTA column, with protein elution using a gradient of imidazole buffers. Eluted protein was analyzed by SDS-PAGE. For inclusion body processing, the pellet was washed, resuspended in refolding buffer (1 mM DTT, 50 mM Tris, 200 mM NaCl, 8 M urea), and stirred at 4°C. DTT and ultrasonic treatment were applied to aid solubilization. After centrifugation, the supernatant was subjected to a second refolding cycle and purified using a Ni-NTA column. Refolding was completed with a solution containing arginine, GSSG, and GSH. The refolded protein was dialyzed and concentrated to 0.2 mg/mL, further purified using a Ni-TED gravity column, yielding the desired *TtHCT1* protein purity.

Enzyme Activity Assay, Specifically, a 200 μL reaction mixture containing 100 mM Tris-HCl (pH 7.5), 5 mM ATP, 0.5 mM Phlorin, 0.5 mM p-Coumaroyl-CoA, and 1 mL of 0.2 mg/mL crude recombinant protein was prepared. The reaction was incubated at 30°C in a water bath for 1 hour. After the reaction, 200 μL of acetonitrile was added to terminate the reaction. The mixture was centrifuged, and the supernatant was collected for HPLC analysis. This procedure was repeated three times for consistency

# **Statistics and reproducibility**

In this study, HiFi and HiC sequencing used for genome assembly were performed as biological replicates once. RNA-seq and metabolomic profiling were each conducted with three biological replicates. For transcript-metabolite network analysis, a correlation coefficient > 0.9, and a p-value < 0.05, were used as screening criteria. Subcellular localization was also performed with three biological replicates. GO and KEGG enrichment analyses (P < 0.05) were carried out using the clusterProfiler package, with multiple testing corrections applied using FDR, and the significance level was set at P < 0.05. All data are expressed as mean ± standard deviation.

**Reference**

Buchfink, B., Xie, C., Huson, et al., 2015. Fast and sensitive protein alignment using DIAMOND. Nat Methods 12, 59–60. https://doi.org/10.1038/nmeth.3176

Burge, C., Karlin, S., 1997. Prediction of complete gene structures in human genomic DNA. J Mol Biol 268, 78–94. https://doi.org/10.1006/jmbi.1997.0951

Cantalapiedra, C.P., Hernández-Plaza, A., Letunic, I., et al., 2021. eggNOG-mapper v2: Functional Annotation, Orthology Assignments, and Domain Prediction at the Metagenomic Scale. Mol Biol Evol 38, 5825–5829. https://doi.org/10.1093/molbev/msab293

Chan, P.P., Lowe, T.M., 2019. tRNAscan-SE: Searching for tRNA Genes in Genomic Sequences. Methods Mol Biol 1962, 1–14. https://doi.org/10.1007/978-1-4939-9173-0_1

Chen, S., Zhou, Y., Chen, Y., et al., 2018. fastp: an ultra-fast all-in-one FASTQ preprocessor. Bioinformatics 34, i884–i890. https://doi.org/10.1093/bioinformatics/bty560

Cheng, H., Concepcion, G.T., Feng, X., et al., 2021. Haplotype-resolved de novo assembly using phased assembly graphs with hifiasm. Nat Methods 18, 170–175. https://doi.org/10.1038/s41592-020-01056-5

Durand, N.C., Shamim, M.S., Machol, I., et al., 2016. Juicer Provides a One-Click System for Analyzing Loop-Resolution Hi-C Experiments. Cell Syst 3, 95–98. https://doi.org/10.1016/j.cels.2016.07.002

Emms, D.M., Kelly, S., 2019. OrthoFinder: phylogenetic orthology inference for comparative genomics. Genome Biol 20, 238. https://doi.org/10.1186/s13059-019-1832-y

Flynn, J.M., Hubley, R., Goubert, C., et al., 2020. RepeatModeler2 for automated genomic discovery of transposable element families. Proc Natl Acad Sci U S A 117, 9451–9457. https://doi.org/10.1073/pnas.1921046117

Gabriel, L., Brůna, T., Hoff, K.J., et al., 2024. BRAKER3: Fully automated genome annotation using RNA-seq and protein evidence with GeneMark-ETP, AUGUSTUS and TSEBRA. bioRxiv 2023.06.10.544449. https://doi.org/10.1101/2023.06.10.544449

Haas, B.J., Salzberg, S.L., Zhu, W., et al., 2008. Automated eukaryotic gene structure annotation using EVidenceModeler and the Program to Assemble Spliced Alignments. Genome Biology 9, R7. https://doi.org/10.1186/gb-2008-9-1-r7

Hu, J., Wang, Z., Sun, Z., et al., 2024. NextDenovo: an efficient error correction and accurate assembly tool for noisy long reads. Genome Biol 25, 107. https://doi.org/10.1186/s13059-024-03252-4

Katoh, K., Standley, D.M., 2013. MAFFT multiple sequence alignment software version 7: improvements in performance and usability. Mol Biol Evol 30, 772–780. https://doi.org/10.1093/molbev/mst010

Keilwagen, J., Hartung, F., Grau, J., 2019. GeMoMa: Homology-Based Gene Prediction Utilizing Intron Position Conservation and RNA-seq Data. Methods Mol Biol 1962, 161–177. https://doi.org/10.1007/978-1-4939-9173-0_9

Kim, D., Langmead, B., Salzberg, S.L., 2015. HISAT: a fast spliced aligner with low memory requirements. Nat Methods 12, 357–360. https://doi.org/10.1038/nmeth.3317

Kumar, S., Suleski, M., Craig, J.M., et al., 2022. TimeTree 5: An Expanded Resource for Species Divergence Times. Mol Biol Evol 39, msac174. https://doi.org/10.1093/molbev/msac174

Langfelder, P., Horvath, S., 2008. WGCNA: an R package for weighted correlation network analysis. BMC Bioinformatics 9, 559. https://doi.org/10.1186/1471-2105-9-559

Li, H., 2018. Minimap2: pairwise alignment for nucleotide sequences. Bioinformatics 34, 3094–3100. https://doi.org/10.1093/bioinformatics/bty191

Loescher, S., Groeer, S., Walther, A., 2018. 3D DNA Origami Nanoparticles: From Basic Design Principles to Emerging Applications in Soft Matter and (Bio-)Nanosciences. Angew Chem Int Ed Engl 57, 10436–10448. https://doi.org/10.1002/anie.201801700

Majoros, W.H., Pertea, M., Salzberg, S.L., 2004. TigrScan and GlimmerHMM: two open source ab initio eukaryotic gene-finders. Bioinformatics 20, 2878–2879. https://doi.org/10.1093/bioinformatics/bth315

Marçais, G., Kingsford, C., 2011. A fast, lock-free approach for efficient parallel counting of occurrences of k-mers. Bioinformatics 27, 764–770. https://doi.org/10.1093/bioinformatics/btr011

Mendes, F.K., Vanderpool, D., Fulton, B., et al., 2021. CAFE 5 models variation in evolutionary rates among gene families. Bioinformatics 36, 5516–5518. https://doi.org/10.1093/bioinformatics/btaa1022

Ou, S., Jiang, N., 2018. LTR_retriever: A Highly Accurate and Sensitive Program for Identification of Long Terminal Repeat Retrotransposons. Plant Physiol 176, 1410–1422. https://doi.org/10.1104/pp.17.01310

Potter, S.C., Luciani, A., Eddy, S.R., et al., 2018. HMMER web server: 2018 update. Nucleic Acids Res 46, W200–W204. https://doi.org/10.1093/nar/gky448

Price, M.N., Dehal, P.S., Arkin, A.P., 2010. FastTree 2--approximately maximum-likelihood trees for large alignments. PLoS One 5, e9490. https://doi.org/10.1371/journal.pone.0009490

Puttick, M.N., 2019. MCMCtreeR: functions to prepare MCMCtree analyses and visualize posterior ages on trees. Bioinformatics 35, 5321–5322. https://doi.org/10.1093/bioinformatics/btz554

Rhie, A., Walenz, B.P., Koren, S., et al., 2020. Merqury: reference-free quality, completeness, and phasing assessment for genome assemblies. Genome Biology 21, 245. https://doi.org/10.1186/s13059-020-02134-9

Riehl, K., Riccio, C., Miska, E.A., et al., 2022. TransposonUltimate: software for transposon classification, annotation and detection. Nucleic Acids Res. 24, 50. https://doi.org/10.1093/nar/gkac136

Roach, M.J., Schmidt, S.A., Borneman, A.R., 2018. Purge Haplotigs: allelic contig reassignment for third-gen diploid genome assemblies. BMC Bioinformatics 19, 460. https://doi.org/10.1186/s12859-018-2485-7

Robinson, J.T., Turner, D., Durand, N.C., et al., 2018. Juicebox.js Provides a Cloud-Based Visualization System for Hi-C Data. Cell Syst 6, 256-258.e1. https://doi.org/10.1016/j.cels.2018.01.001

Sim, S.B., Corpuz, R.L., Simmonds, T.J., et al., 2022. HiFiAdapterFilt, a memory efficient read processing pipeline, prevents occurrence of adapter sequence in PacBio HiFi reads and their negative impacts on genome assembly. BMC Genomics 23, 157. https://doi.org/10.1186/s12864-022-08375-1

Simão, F.A., Waterhouse, R.M., Ioannidis, P., et al., 2015. BUSCO: assessing genome assembly and annotation completeness with single-copy orthologs. Bioinformatics 31, 3210–3212. https://doi.org/10.1093/bioinformatics/btv351

Stamatakis, A., 2014. RAxML version 8: a tool for phylogenetic analysis and post-analysis of large phylogenies. Bioinformatics 30, 1312–1313. https://doi.org/10.1093/bioinformatics/btu033

Sun, P., Jiao, B., Yang, Y., et al., 2022. WGDI: A user-friendly toolkit for evolutionary analyses of whole-genome duplications and ancestral karyotypes. Mol Plant 15, 1841–1851. https://doi.org/10.1016/j.molp.2022.10.018

Tarailo-Graovac, M., Chen, N., 2009. Using RepeatMasker to identify repetitive elements in genomic sequences. Curr Protoc Bioinformatics Chapter 4, 4.10.1-4.10.14. https://doi.org/10.1002/0471250953.bi0410s25

Vurture, G.W., Sedlazeck, F.J., Nattestad, M., et al., 2017. GenomeScope: fast reference-free genome profiling from short reads. Bioinformatics 33, 2202–2204. https://doi.org/10.1093/bioinformatics/btx153

Walker, B.J., Abeel, T., Shea, T., et al., 2014. Pilon: An Integrated Tool for Comprehensive Microbial Variant Detection and Genome Assembly Improvement. PLOS ONE 9, e112963. https://doi.org/10.1371/journal.pone.0112963

Wang, Y., Tang, H., Debarry, J.D., et al., 2012. MCScanX: a toolkit for detection and evolutionary analysis of gene synteny and collinearity. Nucleic Acids Res 40, e49. https://doi.org/10.1093/nar/gkr1293

Wolff, J., Rabbani, L., Gilsbach, R., et al., 2020. Galaxy HiCExplorer 3: a web server for reproducible Hi-C, capture Hi-C and single-cell Hi-C data analysis, quality control and visualization. Nucleic Acids Res 48, W177–W184. https://doi.org/10.1093/nar/gkaa220

Wu, T., Hu, E., Xu, S., et al., 2021. clusterProfiler 4.0: A universal enrichment tool for interpreting omics data. Innovation (Camb) 2, 100141. https://doi.org/10.1016/j.xinn.2021.100141

Xu, M., Guo, L., Gu, S., et al., 2020. TGS-GapCloser: A fast and accurate gap closer for large genomes with low coverage of error-prone long reads. Gigascience 9, giaa094. https://doi.org/10.1093/gigascience/giaa094

Zhang, R., Li, G., Wang, X., et al., 2022. TEsorter: an accurate and fast method to classify LTR-retrotransposons in plant genomes. Hortic Res 9, uhac017. https://doi.org/10.1093/hr/uhac017

Zhang, X., He, C., Ye, B., et al., 2017. Optimization and quality control of genome-wide Hi-C library preparation. Yi Chuan 39, 847–855. https://doi.org/10.16288/j.yczz.17-152
